# Titanium Oxycarbide as Platinum-Free Electrocatalyst for Ethanol Oxidation

**DOI:** 10.1021/acscatal.3c04097

**Published:** 2023-12-18

**Authors:** Niusha Shakibi Nia, Christoph Griesser, Thomas Mairegger, Eva-Maria Wernig, Johannes Bernardi, Engelbert Portenkirchner, Simon Penner, Julia Kunze-Liebhäuser

**Affiliations:** † Institute of Physical Chemistry, University of Innsbruck, 6020 Innsbruck, Austria; ‡ USTEM, Technische Universität Wien, Stadionalle 2, 1020 Wien, Austria

**Keywords:** titanium oxycarbide, compound material, DEMS, EOR, energy conversion, platinum-free

## Abstract

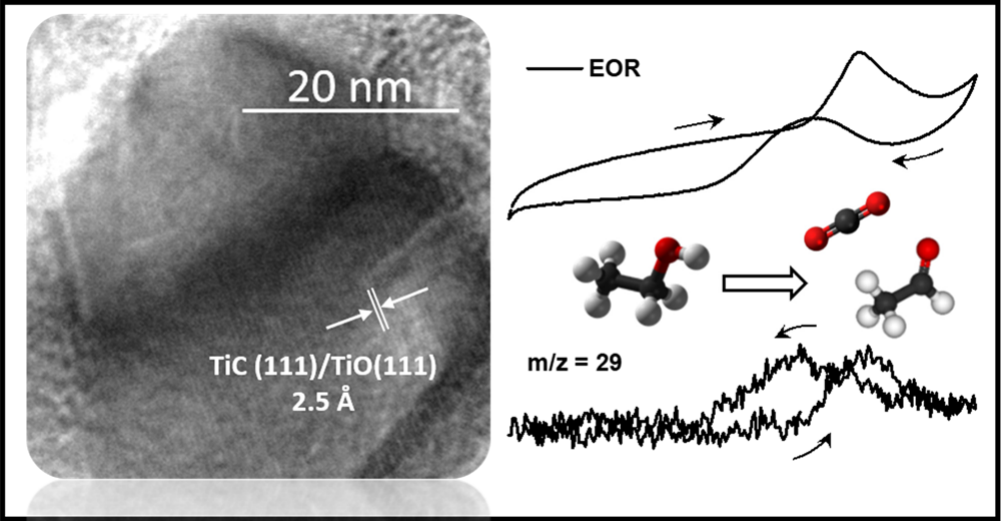

The compound material titanium oxycarbide (TiOC) is found to be an effective electrocatalyst for the electrochemical oxidation of ethanol to CO_2_. The complete course of this reaction is one of the main challenges in direct ethanol fuel cells (DEFCs). While TiOC has previously been investigated as catalyst support material only, in this study we show that TiOC alone is able to oxidize ethanol to acetaldehyde without the need of expensive noble metal catalysts like Pt. It is suggested that this behavior is attributed to the presence of both undercoordinated sites, which allow ethanol to adsorb, and oxygenated sites, which facilitate the activation of water. This is a milestone in DEFC research and development and opens up innovative possibilities for the design of catalyst materials for intermediate temperature fuel cells.

Alternative energy conversion systems are considered crucial for mitigating challenges related to limited fossil fuel supplies and a rapid transition toward the implementation of renewable energies.^1^ While electrification is considered to be the method of choice for most applications, aircraft, ships, and even trucks will most likely continue to run mainly on carbon-neutral, synthetic solar fuels derived by hydrogenation of CO_2_.^2^ Accordingly, alternative energy scenarios are outlined, which may become critical for addressing climate change, while at the same time they will likely result in trillions of dollars in net energy cost savings.^3,4^

Ethanol is one of the most promising alternatives to gasoline and can be obtained via solar-energy-driven conversion of biomass to bioethanol that offers economy, environment, and energy benefits.^5^ Accordingly, direct ethanol fuel cells (DEFCs) are regarded as important energy conversion devices to power mobile and portable applications and have been subject to numerous studies in recent years.^6,7^

The anodic reaction in DEFCs, i.e., the ethanol oxidation reaction (EOR), is a multielectron transfer process that suffers from slow kinetics of the ethanol electrooxidation reaction and its poor selectivity toward complete oxidation to CO_2_.^8,9^ Up to now, mainly Pt- and Pd-based electrocatalysts have been employed for the EOR in DEFCs, which has hampered their economic success because of the high costs of the noble metals.^10,11^ The highest faradaic efficiency was, to the best of our knowledge, achieved by a SnO_2_-Rh nanosheet catalyst, which demonstrated a 72.8% conversion rate of ethanol to CO_2_ at 0.78 V_RHE_ in an alkaline electrolyte.^12^ Under acidic conditions, a PtAuSn/W_2_C catalyst achieved the highest Faraday efficiency with 6.5% at 0.65 V_RHE_.^13^ Various studies have suggested and proved that efficient EOR with low ethanol concentrations requires the use of elevated temperatures > 60 °C.^9,14,15^ This causes issues concerning the stability and durability of the catalyst materials that are usually mainly based on carbon-supported Pt (alloys). Carbon is prone to corrosion under conventional DEFC operation conditions, which can result in detachment and agglomeration of catalyst nanoparticles.^16^ Higher efficiencies in DEFCs, specifically higher selectivity toward complete oxidation to CO_2_, could potentially initiate a paradigm change in the use of this technology because it might enable the operation at lower temperatures, which leads to milder fuel cell operation conditions.

The central reason for the slow kinetics and low efficiency toward full oxidation to CO_2_ is the complexity of the electrochemical EOR that has been thoroughly mechanistically described in the past decades.^8,17,18^ The EOR consists of multiple steps with a number of adsorbed intermediates and side products.^19,20^ The total oxidation to CO_2_ proceeds via two types of adsorbed intermediates, C1_ad_ and C2_ad_, which are fragments with one and two carbon atoms. To elucidate the mechanism of the EOR in acidic environment, differential electrochemical mass spectrometry (DEMS),^15,21−25^ Fourier transform infrared spectroscopy (FTIR),^20,25−29^ gas chromatography (GC), high-performance liquid chromatography (HPLC),^30−32^ and liquid-state nuclear magnetic resonance spectroscopy (NMR)^33^ have been employed.

The higher structural and electronic complexity of compound materials offers equivalent possibilities to engineer adsorption sites and thereby break the known metal scaling relations,^34,35^ as through promotion or alloying of metal electrodes. PtRu and PtSn are the best investigated solid solutions for the EOR. In the case of PtRu, intermediates are found to be more weakly adsorbed, and the dissociative adsorption of ethanol seems to be inhibited by Pt, while Ru seems to enhance the oxidation of strongly bound adsorbed intermediates through the activation and dissociation of water to give a higher relative yield of CO_2_ than on pure Pt.

It has been theoretically shown that the presence of novel interface sites between two materials with different scaling properties offers unique possibilities for bifunctional activity gains.^36,37^ Furthermore, oxygenated titanium species improve the CO tolerance of platinum because of their ability to supply oxygenated species (OH) at low potentials.^38^ This bifunctional mechanism was proposed in the case of TiC-supported Pt catalysts to explain their enhanced activity toward the methanol oxidation reaction (MOR).^38−40^ In case of the EOR studied in the present paper, the oxidation of adsorbed intermediates, such as CO, CH_*x*_, and CH_*x*_O (all C1 species), and of acetaldehyde through the activation of water forming adsorbed OH at the surface is key to forming CO_2_ via the so-called C1 pathway.^33^ The activation of H_2_O is significantly enhanced on the titanium oxycarbide (TiOC) support because it promotes H_2_O dissociation and surface oxidation.^41,42^

Titanium oxycarbide (TiOC) has been shown to be suited as a stable and synergistic support for Pt nanoparticles during the EOR in acidic electrolytes.^41^ A significant enhancement of the CO_2_ efficiency was observed in combination with a surface passivation of the TiOC support and with respect to the reference catalyst material Pt/C Vulcan. The difference in CO_2_ efficiency during the EOR and the enhanced oxidation activity toward adsorbed CO was found to be related to the presence of TiO_2_ and OH groups on the oxycarbide support,^41^ which improve the CO tolerance of the Pt catalyst through this bifunctional mechanism.^43,44^ However, the potential catalytic activity of the TiOC support material, itself, has not been anticipated or reported to date.

In this work, we report on the performance of the compound material TiOC as an electrocatalyst for the electrochemical oxidation of ethanol without the addition of any noble metal catalyst. DEMS and subtractively normalized interfacial Fourier transform infrared spectroscopy (SNIFTIRS) reveal the formation of acetaldehyde via online and in situ detection during electrocatalytic EOR operation. Prior to the electrocatalytic and electroanalytic evaluation of the compound material, the morphology and structure of the TiOC are thoroughly studied with transmission electron microscopy (TEM). Figure 1 reveals individual differently oriented grains displaying strong diffraction contrast (black-and-gray areas) in the overview bright field image (Figure 1a). The selected area electron diffraction pattern (inset in Figure 1a) can be explained by the presence of TiC and/or TiO [khamrabaevite TiC (face-centered cubic structure, *a* = 4.325 Å) or cubic TiO (face-centered cubic structure, *a* = 4.117 Å)]. The presence of TiOC is inferred through the combination of the structural with the compositional data, shown in Figure 1b, that reveals the homogeneous distribution of carbon, oxygen, and titanium. Most of the grains reveal distinct particle outlines, thereby indicating defined morphologies. The observed particle shapes are characteristic for projected truncated pyramidal particles in [100] or [110] direction,^45^ which—because of the well-defined particle outlines and the strong diffraction contrast in the respective dark-field images (Figure 1c)—directly reveal their three-dimensional truncated pyramidal shape by exposing the characteristic contour lines.^46^ Finally, Figure 1d confirms the presence of the TiO/TiC material by revealing (111) lattice planes of TiOC [experimentally, 2.5 Å; fcc khamrabaevite TiC (theoretical spacing, 2.49 Å) or fcc TiO (2.41 Å)].

**Figure 1:**
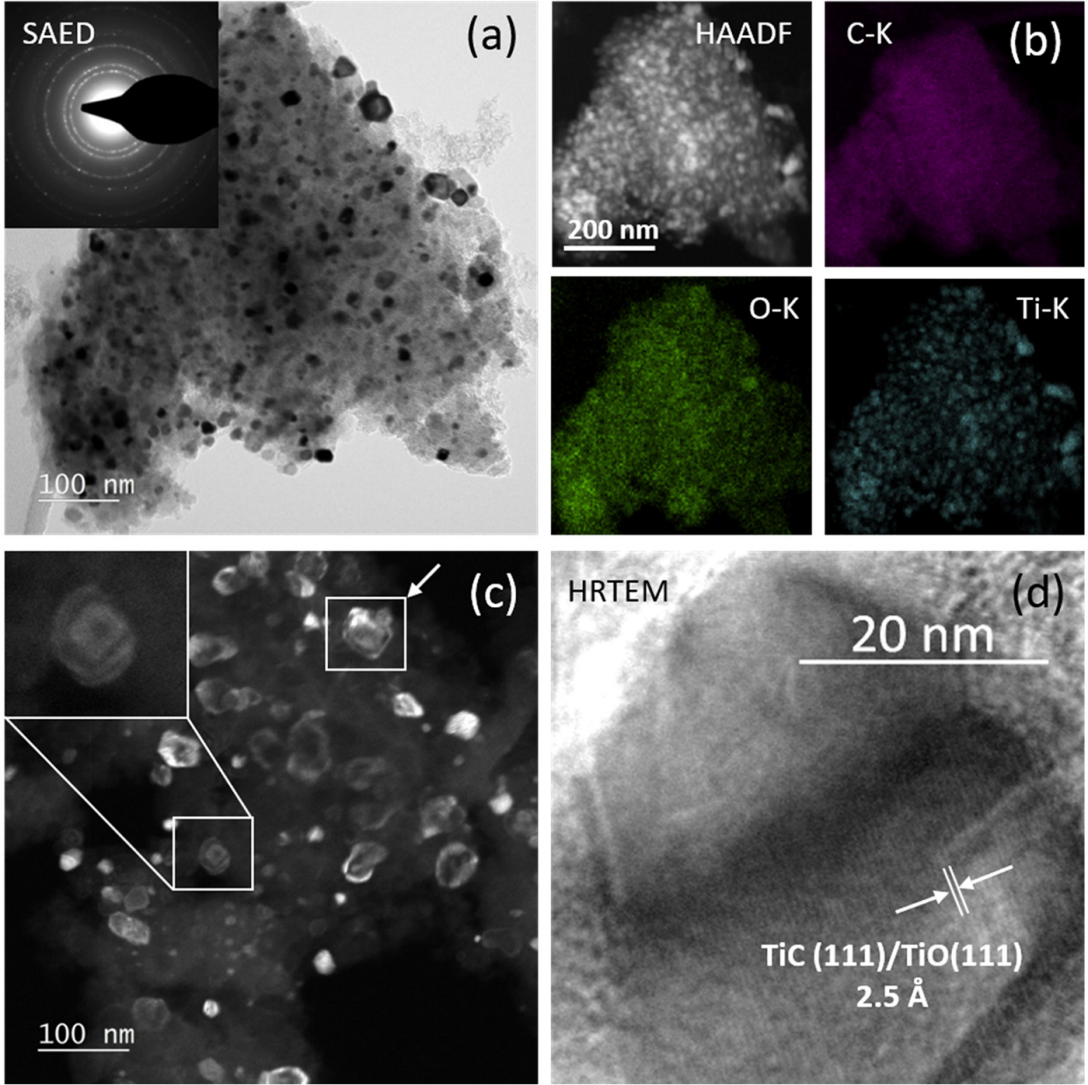
Transmission electron microscopy (TEM) images of the TiOC powder material. (a) Overview bright-field image with the corresponding selected area electron diffraction (SAED) pattern (inset). (b) High-angle annular dark-field (HAADF) image (top left) and individual EDX maps of the C (top right), O (bottom left), and Ti K-edges (bottom right). (c) Dark-field image of the TiOC powder directly showing diffraction contrast caused by differently oriented grains. For two selected individual grains (boxes), contour lines of equal height are clearly visible. (d) High-resolution (HR) TEM image of a TiOC grain revealing TiC/TiO (111) lattice fringes.

The stability range of TiOC under anodic polarization is limited by the formation of TiO_2_ and CO_2_ at potentials > 0.9 V_SHE_.^41,47^ The CO_2_ evolution without ethanol in the electrolyte can be tracked online with DEMS to verify this and to set the background for the EOR studies (see Figure S1 in the Supporting Information). It has, however, previously been found that TiOC corrosion is delayed in the presence of ethanol in the electrolyte. Ethanol molecules adsorb on the TiOC surface and inhibit its oxidation, which is also expected to favor the EOR in the presence of suitable catalyst nanoparticles supported on TiOC.^47^ Therefore, the stability range is likely larger toward more anodic potentials under the EOR operation. DEMS is further employed to qualitatively identify the reaction products forming during the EOR in 0.5 M H_2_SO_4_ and 0.1 M EtOH at 20 °C (see Figure 2). Potentiodynamic EOR studies enable qualitative and online identification of the reaction products through the occurrence of specific ionic signals. The formation onsets are determined at the potential where the respective ionic currents deviate from the background signal (see Figure S2 in the Supporting Information for details). Acetaldehyde formation is visible through the occurrence of a signal at *m*/*z* = 29 (CHO^+^) at around 0.6 V_SHE_, which is well in line with the ionic current observed upon potential cycling, where a slight increase in the *m*/*z* = 29 signal can be observed at 0.6 V_SHE_ (Figure 2b). The very subtle deviation from the background at *m*/*z* = 22 (CO_2_^+2^) even indicates the formation of very small amounts of CO_2_ at the same onset potential. Prior to these EOR investigations, the TiOC was preconditioned by measuring a blank CV (see Figure S3a in the Supporting Information) between 0.03 V_SHE_ and 1.1 V_SHE_ followed by three CO stripping sweeps at 2 mV s^–1^ (see Figure S3b in the Supporting Information).

**Figure 2:**
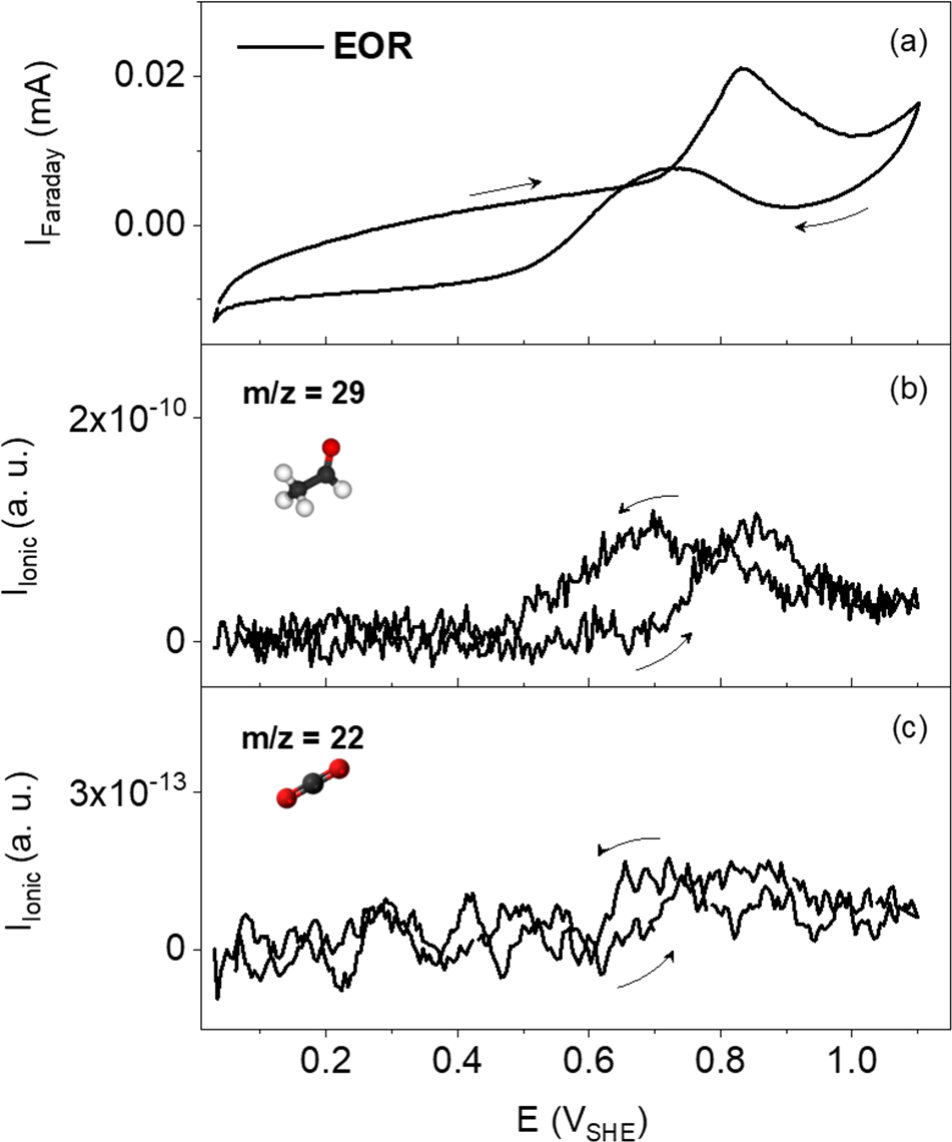
EOR of TiOC powder ink. (a) CV recorded in 0.5 M H_2_SO_4_ and 0.1 M EtOH at 20 °C and mass spectrometric cyclic voltammograms (MSCVs) for (b) *m*/*z* = 29 and (c) *m*/*z* = 22. Scan rate: 2 mV s^–1^. Specific gravimetric peak current at 0.83 V_SHE_: 0.01 mA mg^–1^.

SNIFTIR spectra were recorded at increasing anodic potentials from 0.3 V_SHE_ to 1.0 V_SHE_ (Figure 3). The formation of acetaldehyde (dashed gray boxes) starts at 0.7 V_SHE_. The band at 3080–2650 cm^–1^ (C–H stretching mode) and at 1750–1530 cm^–1^ (C=O vibrational mode) correspond to the vibrational modes of the aldehyde group, which overlaps with the stretching and bending modes of the methyl group in the acetaldehyde.^48^ The bands at 2360 cm^–1^ and 2331 cm^–1^ are assigned to gaseous CO_2_ (R and P branch),^49^ which arise because of residual ambient atmosphere in the beam path. Significantly, at 1.0 V_SHE_, the spectral profile undergoes a notable transformation characterized by the emergence of upward features. These features manifest as a band at 1640 cm^–1^, associated with the bending mode of water, and a broad band in the range 3100–3700 cm^–1^. This wave exhibits prominent peaks at 3100 cm^–1^ and 3635 cm^–1^, which can be assigned to the stretching vibration of water and the Ti-OH, respectively.^50^ The reason for this can be attributed to the substantial reduction in surface OH groups (Ti-OH) stemming from the oxidation of TiOC and the simultaneous decrease in the water content resulting from the oxidation reaction.

**Figure 3:**
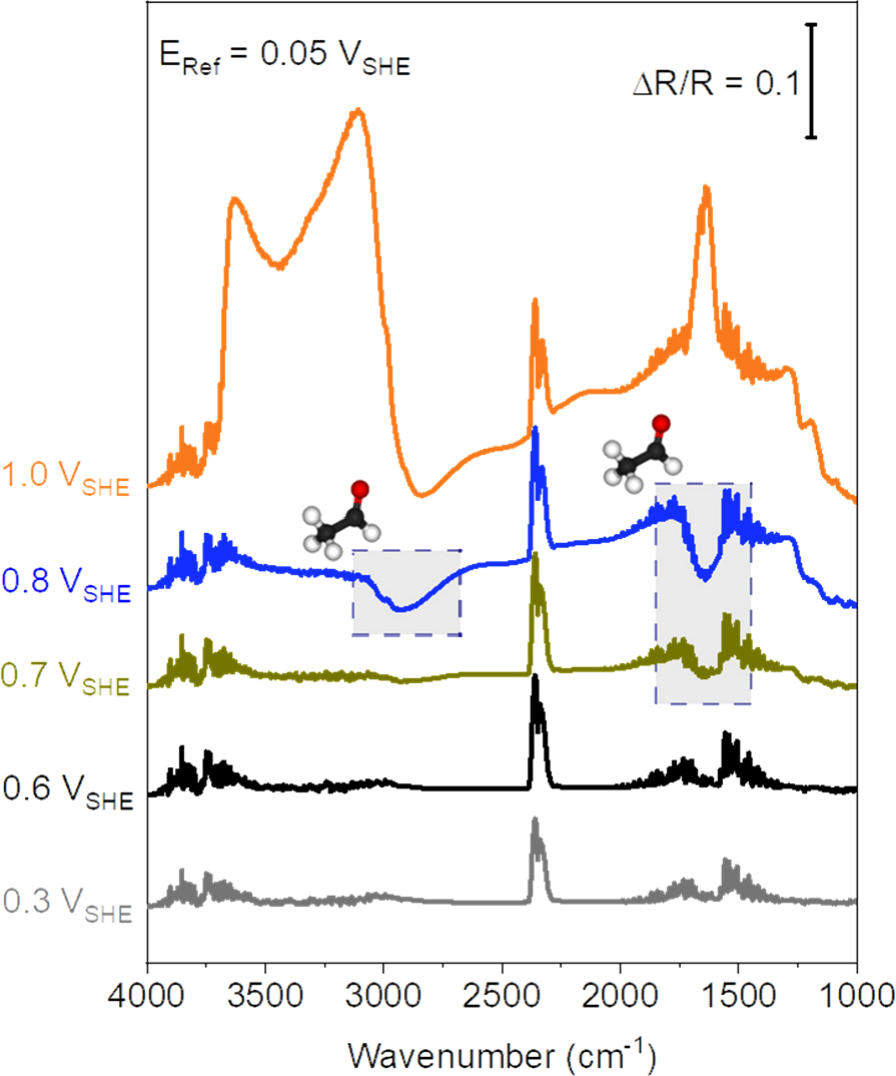
SNIFTIR spectra at increasing anodic potentials from 0.3 V_SHE_ to 1.0 V_SHE_ recorded in 0.5 M H_2_SO_4_ and 1 M EtOH. The bands, indicating acetaldehyde formation, begin to grow at a potential of ∼0.7 V_SHE_ (dashed gray boxes). The reference spectrum was taken at 0.05 V_SHE_ .

For further evaluation of the CO_2_ conversion efficiency at TiOC, current transients of the EOR are recorded together with the corresponding DEMS signals for the *m*/*z* ratios of 29 and 22 (Figure S4).

High-resolution XPS spectra (Ti 2p and C 1s regions) of the TiOC electrodes have been recorded after the SNIFTIRS studies to confirm the continuous presence of carbide and, consequently, the long-term stability of the TiOC material (Figure S5). Moreover, analysis of the Ti 2p region implies that, in addition to the species associated with TiO_2_, TiC, and TiOC, reduced TiO_2–*x*_ species are present, which signifies the existence of oxygen vacancies. This conclusion gains further support from Raman experiments conducted on the powder catalyst (Figure S6). TiOC is known to decompose both thermodynamically and at contact with ambient air to TiO_2_ and carbon at its outermost surface.^51^ At potentials larger than 0.9 V_SHE_, where more TiO_2_ and carbon are formed at the solid/liquid interface, TiOC starts to passivate. In the presence of ethanol, however, this passivation is partly inhibited, which has been suggested to be caused by ethanol adsorption at the TiO_2_-terminated oxycarbide surface.^47^ Therefore, we propose the following pathway for the electrooxidation of ethanol on this material, which is, at the current stage of investigation, highly speculative: initially, ethanol adsorbs on the catalyst surface, preferably binding to undercoordinated sites, such as oxygen vacancies.^52^ It is well known that TiO_2_ sites, which are inherently present on the surface because of partial oxidation of the TiO_2–*x*_ sites, can supply oxygenated species (−OH groups) through their ability to dissociate the water from the electrolyte. These OH groups can further react with the adsorbed ethanol to form acetaldehyde. The combination of the present −OH groups and the abundance of oxygen vacancies resulting from the composite nature of the material (see Figure 1) might account for the observed exceptional catalytic activity.

In this work, the electrocatalytic activity of TiOC toward the EOR is investigated under acidic electrolyte conditions at room temperature via DEMS and SNIFTIRS investigations. TEM analysis reveals three-dimensional truncated pyramidal-shaped TiOC particles, and EDX and XPS confirm the stability of the TiOC material under both electrochemical conditions and exposure to ambient atmosphere. We show, for the first time, that TiOC is able to oxidize ethanol to acetaldehyde without the need for expensive noble metals. The catalytic activity is attributed to the presence of both undercoordinated sites for ethanol adsorption and oxygenated sites facilitating the oxidation of adsorbed ethanol. While it is unlikely that TiOC becomes a valuable catalyst by itself, TiOC may serve as an important cocatalyst and catalyst support simultaneously, thereby adding improved activity to the system by enabling bifunctional activity gains.

## Supporting Information Available

Experimental methods, TiOC powder ink blank measurement (Figure S1), exemplary onset determination (Figure S2), blank CV measurements and CO stripping (Figure S3), current transients and corresponding mass spectrometric ionic current signals (Figure S4), high-resolution XP spectra (Figure S5) and Raman spectra of the TiOC powder catalyst (Figure S6) (PDF)
