# Supplementary material for: Titanium Oxycarbide as Platinum-Free Electrocatalyst for Ethanol Oxidation
Source: ACS Catal. 2023 Dec 18;14(1):324–9. doi: 10.1021/acscatal.3c04097 (PMC10775143; doi:10.1021/acscatal.3c04097)
Supplement: Supplementary file 1 — cs3c04097_si_001.pdf [file cs3c04097_si_001.pdf]

Supporting Information: Titanium Oxycarbide as Platinum-free Electrocatalyst for Ethanol Oxidation

Niusha Shakibi Nia,[a] Christoph Griesser,[a] Thomas Mairegger,[a] Eva-Maria Wernig,[a] Johannes Bernardi,[b] Engelbert Portenkirchner, [a,*] Simon Penner,[a] and Julia Kunze-Liebhäuser [a,*]

a) Institute of Physical Chemistry, University of Innsbruck, 6020 Innsbruck, Austria
b) USTEM, Technische Universität Wien, Stadionalle 2, 1020 Wien, Austria

* Engelbert Portenkirchner − Department of Physical Chemistry, University of Innsbruck, Innsbruck 6020, Austria; orcid.org/0000-0002-6281-5243; Email: Engelbert.Portenkirchner@uibk.ac.at
* Julia Kunze-Liebhäuser − Department of Physical Chemistry, University of Innsbruck, Innsbruck 6020, Austria; orcid.org/0000-0002-8225-3110; Email: Julia.Kunze@uibk.ac.at

Materials and Methods

Materials Synthesis and Electrode Preparation: The titanium oxycarbide powder was synthesized following a previously published procedure.[1] The inks were prepared by mixing 2 mg of each powder with 15 mL of Nafion solution (5 wt %, Sigma Aldrich) and 0.5 mL of de-ionized (DI) water (Merck, Germany, Milli-Q 18.2MU). To ensure a good dispersion of the powder in the solution, the ink was sonicated for 20 to 30 minutes. A glassy carbon (GC) disk (Sigradur G, Hochtemperatur Werkstoffe GmbH, Germany) with a thickness of 5 mm, a diameter of 10 mm and, for DEMS measurements with a hole of 4 mm diameter in the middle, was used as support for the powder inks. TiOC thin films for SNIFTIRS measurements were prepared by carbothermal reduction of electropolished TiO_2. Therefore, the sample was heated at 8 K/minute in a quartz tube furnace up to 750 °C under 200 sscm Ar flow, followed by 60 minutes of dwelling. Then, 1 sscm C_2H_2 was introduced for 5 minutes followed by another dwelling period of 60 minutes in Ar. The sample was cooled down under an Ar flow of 200 sscm.

Electrochemical Measurements: Cyclic voltammetry was performed at room temperature in a standard glass cell using a three-electrode cell configuration and an Autolab PGSTAT 302 N potentiostat (Nova 1.10 software). Cyclovoltammograms (CVs) were recorded between 0.03 and 1.1 V at 2 mV s^{-1} scan rate (20 cycles). The reference electrode (RE) was a Hg/Hg_2SO_4 (0.1M H_2SO_4) electrode with a potential of 0.66 V versus the standard hydrogen electrode (SHE), and a carbon rod (Ultra carbon corporation, USA, ultra "F" purity) was used as the counter electrode (CE). All potentials are given with respect to the SHE. Prior to each measurement, the oxygen was removed by purging the electrolyte with Argon

for 30 minutes. All the electrolytes were prepared using high purity 18 MΩ deionized (DI) water (Milli-Q, 18.2 MΩ, Millipore, Merck).

Differential Electrochemical Mass Spectrometry (DEMS): DEMS measurements were performed using a Hiden HPR-40 mass spectrometer in an online configuration with the electrochemical cell. In this configuration, the inlet of the capillary was covered by a Teflon membrane (Gore-Tex, 75 µm thick, 50% porosity, 0.02 mm pore diameter) and was placed through a hole on the back side of the GC. 5 mL of the powder ink were deposited on the GC and the membrane. The GC (equipped with the capillary, the membrane and the powder ink) was used as the working electrode (WE) in a hanging meniscus configuration.[2] The applied electron energy for ionization of all species was 70 eV and the used emission current was 500 mA. The secondary electron multiplier (SEM) energy was set to 850 V for detection of all species. Current transient measurements were recorded at 0.60, 0.70, 0.80, 0.85 and 0.90 V for 900 s each.

Transmission Electron Microscopy (TEM): For all TEM analyses, an FEI Tecnai F20 S-TWIN high-resolution analytical transmission electron microscope operated at 200 kV was used. An EDAX Apollo XLT2 silicon-drifted detector was employed to collect the energy-dispersive X-ray (EDX) spectra of the Ti K-, O K- and C K-edges. All samples were supported on holey carbon films without further treatment.

Subtractively Normalized Interfacial Fourier Transform Infrared Spectroscopy (SNIFTIRS): The SNIFTIRS studies were carried out using a VERTEX 70v spectrometer (Bruker) with an additional external chamber (XSA, Bruker), equipped with a liquid N_2 cooled mercury cadmium telluride (MCT) photodetector. A thin-layer cell configuration was employed to minimize the contribution of the electrolyte solution. The IR beam entered the spectro-electrochemical cell through a linear polarizer (Edmund Optics) and with the use of a movable gold-coated mirror. After passing a CaF_2 hemisphere constituting the bottom of the homemade spectro-electrochemical cell, the IR beam was reflected from the surface of the WE that was pressed against the hemisphere and entered the detector with the use of a second gold-coated mirror. The cell was equipped with the TiOC thin film WE, the above-mentioned carbon rod CE and the Hg/Hg_2SO_4 (0.1M H_2SO_4) RE. The strong absorption of the thin electrolyte film was accounted for by subtracting and normalizing the single spectra recorded at a specific applied potential (E_S) with the single spectrum at a specific reference potential (E_R = 0.05 V_{SHE}), at which no reaction should occur, according to equation 1:

$$\Delta R/R = \frac{R(E_S) - R(E_R)}{R(E_R)} \qquad \text{eq. 1.}$$

with $R(E_S)$ being the reflectance sample single spectra and $R(E_R)$ being the reflectance reference single spectra. After normalization, each upward or downward facing band corresponds to consumed/disappeared or formed/accumulated species at the electrode surface, respectively. SNIFTIR

spectra were recorded in a step potential modulation, in which the reference spectrum was acquired at the beginning and then the potentials were stepped more positively for each subsequent spectrum.

X-ray photoelectron spectroscopy (XPS): For characterization of the electrodes' surface chemistry, X-ray photoelectron spectroscopy (XPS) was performed on a MultiLab 2000 (ThermoFisher Scientific), utilizing a monochromatic Al K_α X-ray source (1486.6 eV) and a hemispherical energy analyzer (Thermo Fisher Scientific, Alpha 110). High-resolution (HR) spectra of the regions of interest (Ti 2p, C 1s) were recorded at a pass energy of 25 eV and an energy step size of 0.01 eV. For the fitting of the C 1s region a Shirley type background was used.

Raman spectroscopy: Raman spectra were taken using a WITec Confocal alpha 300 R Raman Imaging Microscope, including a laser of a wavelength of 532 nm and a maximal power output of 20 mW, with a ZEISS LD Plan-NEOFLUAR/40×-objective. The spectra were collected using an 600 g/mm grating and a charged coupled device (CCD)-camera.

Results and Discussion

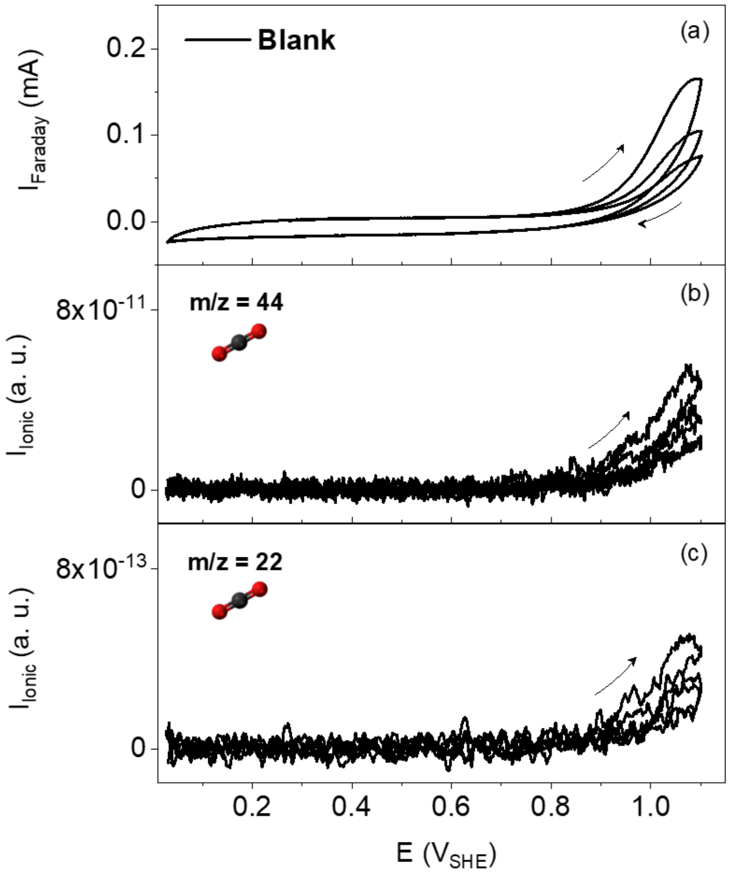

Figure S1: DEMS measurement of a TiOC powder ink reference. (a) CV recorded in 0.5 M H_2SO_4 at 20 °C and MSCVs for (b) m/z = 44 and (c) m/z = 22. Scan rate: 2 mV s^{-1}.

Figure S1 shows blank potentiodynamic DEMS measurements (CV and the corresponding mass spectrometric cyclo-voltammograms (MSCVs)) of the TiOC powder ink electrodes in 0.5 M H_2SO_4 without EtOH. Apart from the CO_2 evolution starting at potentials lager than 0.9 V_{SHE},[1] visible in the ionic DEMS signal at a mass to charge (m/z) ratio of 44 and 22 (CO_2^{++}), respectively (**Figure S1 b,c**), no product formation could be observed.[3]

Onset determination: For the onset determination, a horizontal line through the y-axis zero was drawn in the mass spectrometric linear sweep voltammograms for each recorded *m/z*-signal. When no reaction occurs, the recorded ionic currents fluctuate around this zero line. The onset is determined as that potential, where the linear fit of the signal intersects the zero line. In **Figure S2**, an exemplary onset determination for the DEMS signals for m/z ratio of 29 is given.

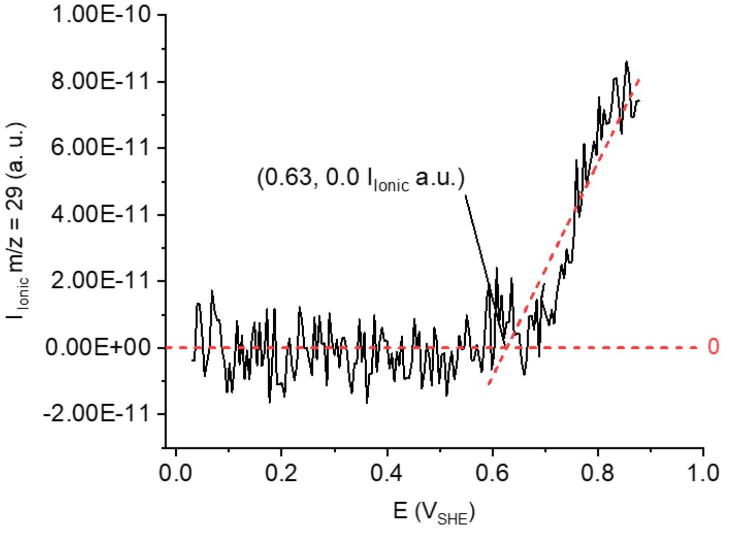

Figure S2: Exemplary onset determination for the DEMS signals with a m/z ratio of 29.

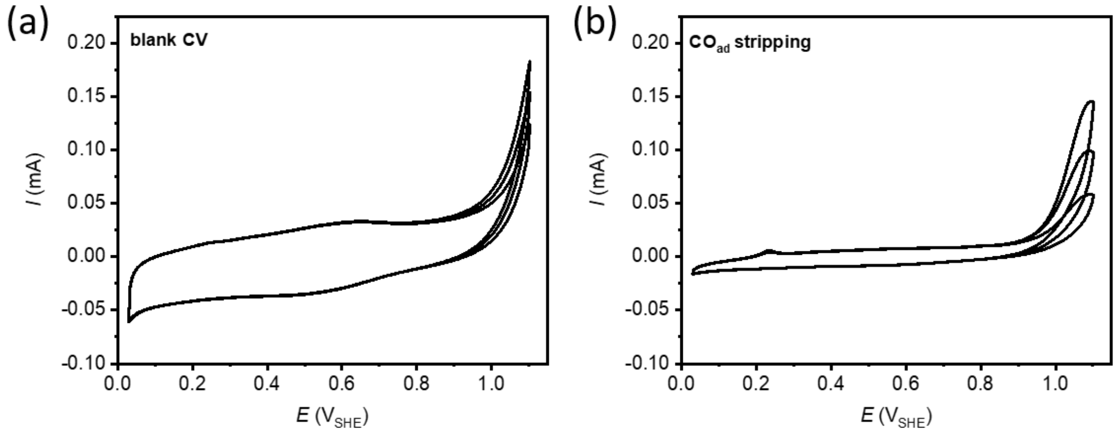

Figure S3: a) Blank CV measurement of a TiOC powder ink between 0.03 and 1.1 V, followed by b) three CO stripping sweeps at 2 mV s^{-1}, recorded in 0.5 M H_2SO_4.

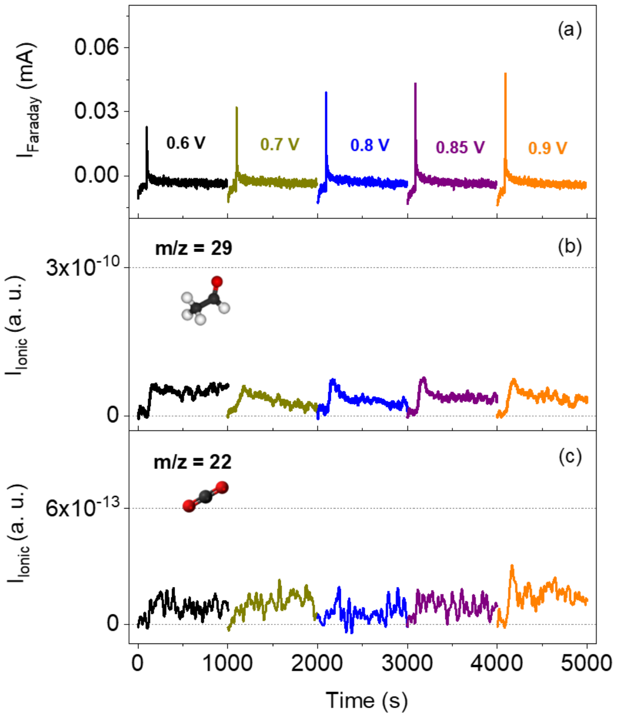

Figure S4: (a) Current transients and corresponding mass spectrometric ionic current signals for (b) m/z = 29 and (c) m/z = 22 of TiOC powder ink recorded in 0.5 M H_2SO_4 and 0.1 M EtOH.

For further evaluation of the CO_2 conversion efficiency at TiOC, and to exclude any kinetic issues due to potential cycling, current transients of the EOR are recorded after stepping the potential from an initial value of 0.05 V to the anodic potentials of interest that ranged from 0.6 V_{SHE} to 0.9 V_{SHE}. The corresponding DEMS signals for m/z ratio of 29 and 22 are simultaneously recorded and shown for the relevant potentials in **Figure S4**. From this one can directly observe that significant acetaldehyde formation takes place at potentials as early as 0.6 V_{SHE} (middle panel), whereas significant deviation of the signal related to CO_2 formation from the background (bottom panel) is only well observed at 0.9 V. At these high anodic potentials, oxidation of the TiOC takes place. This strongly indicates that the TiOC catalysts oxidizes EtOH towards acetaldehyde, however the further oxidation seems to be inhibited as only small amounts of CO_2 (close to the detection limit of the instrument, compare Figure 2, bottom panel) are formed.

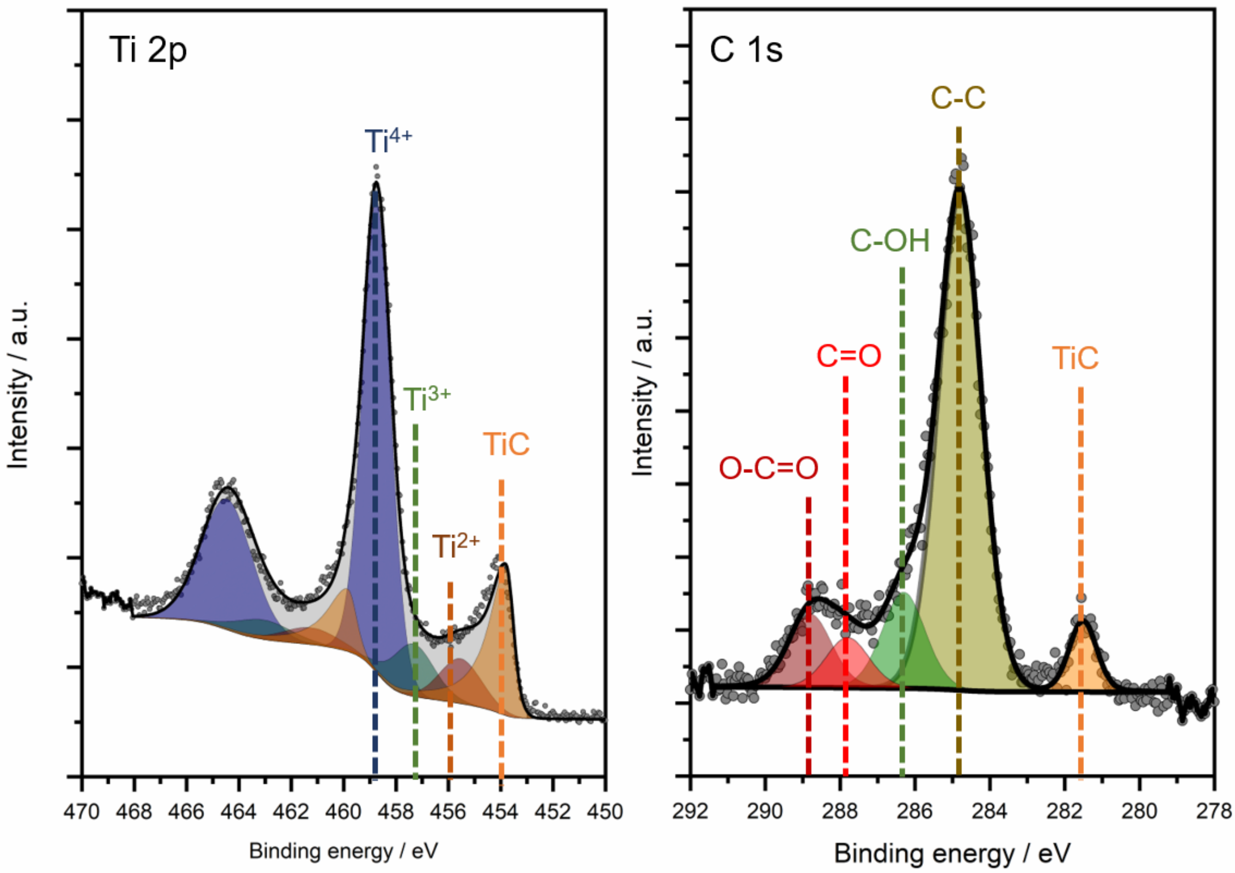

Figure S5: High-resolution XP spectra of the a) Ti 2p and b) C 1s region, respectively. Spectra of an aged TiOC electrode after SNIFTIRS measurements (Figure 3, main text) are recorded. Fitting of the Ti 2p region was performed according to reference [4].

After the electrochemical measurements, an XPS analysis was conducted to investigate the surface chemistry of the thin film electrode. In both the Ti 2p and C 1s regions, distinctive features associated with TiC are detected. In the Ti 2p region, aside from the contribution of Ti^{4+} (i.e. TiO_2), there are observable contributions from further reduced (suboxide) Ti species (Ti^{3+} and Ti^{2+}). This strongly points to the existence of substoichiometric TiO_{2-x} and to the presence of oxygen vacancies at the electrode surface.

Furthermore, the presence of TiC after the electrochemical experiments provides compelling evidence that the material remains stable under operation conditions. This aligns seamlessly with findings in a previous publication (reference 1 in the main manuscript).

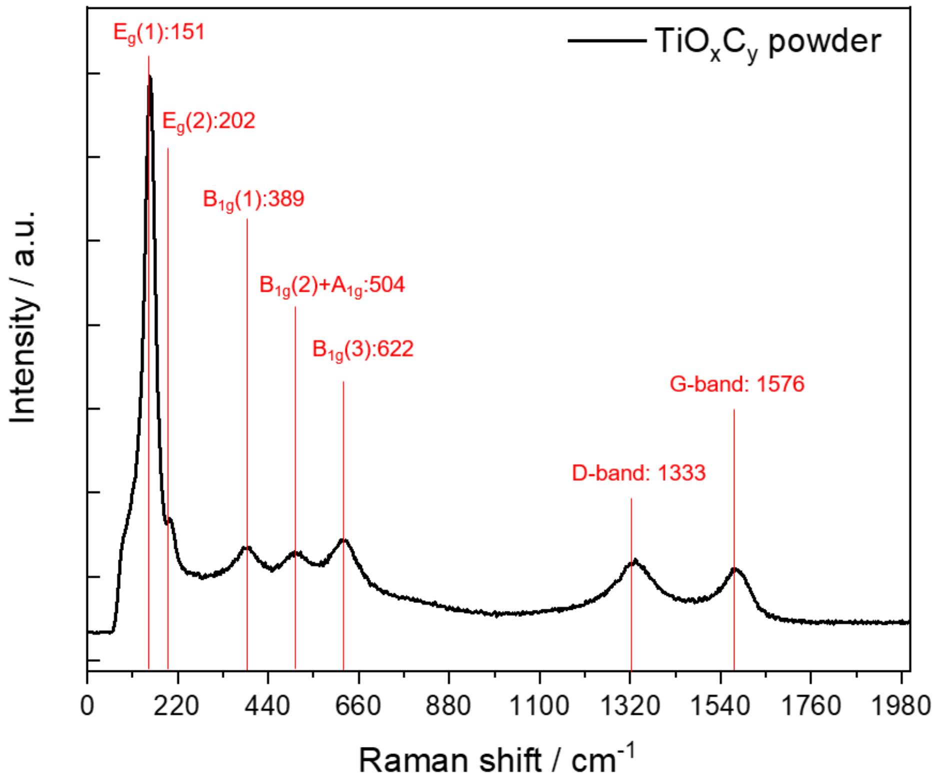

Figure S6: Raman spectra of the TiOC powder catalyst. The vertical red lines mark the wavenumbers of the peak maxima with their assignments.

The Raman spectrum of the TiOC powder reveals noteworthy characteristics. Specifically, the $E_g(1)$ peak exhibits a maximum around 151 cm^{-1}, showing a noticeable shift towards higher wavenumbers when compared to pure TiO_2 (anatase), where the peak is typically around 145 cm^{-1}.[5] The $E_g(2)$ peak at approximately 202 cm^{-1} shows a slight blue shift towards higher wavenumbers .[5] The peak maxima of the $B_{1g}(1)$, $B_{1g}(2)+A_{1g}$, and $B_{1g}(3)$ modes are slightly lower in comparison to pure TiO_2 (anatase).[5]

What is especially intriguing is the resemblance of the Raman spectrum to the spectra of TiO_{2-x} anatase, as previously presented in ref. [6]. This earlier work emphasized the significance of oxygen vacancies in relation to charge storage in sodium-ion batteries. This correspondence strongly suggests the presence of oxygen vacancies in the TiOC powder, which is well in line with the XPS data as depicted in Figure S5. The D and G bands are related to carbon.

Supporting Literature

(1) Calvillo, L.; García, G.; Paduano, A.; Guillen-Villafuerte, O.; Valero-Vidal, C.; Vittadini, A.; Bellini, M.; Lavacchi, A.; Agnoli, S.; Martucci, A.; Kunze-Liebhäuser, J.; Pastor, E.; Granozzi, G. Electrochemical Behavior of TiO x C y as Catalyst Support for Direct Ethanol Fuel Cells at Intermediate Temperature: From Planar Systems to Powders. *ACS Appl. Mater. Interfaces* **2016**, *8* (1), 716–725. https://doi.org/10.1021/acsami.5b09861.

(2) Guillén-Villafuerte, O.; García, G.; Arévalo, M. C.; Rodríguez, J. L.; Pastor, E. New Insights on the Electrochemical Oxidation of Ethanol on Carbon-Supported Pt Electrode by a Novel Electrochemical Mass Spectrometry Configuration. *Electrochem. commun.* **2016**, *63*, 48–51. https://doi.org/10.1016/j.elecom.2015.12.007.

(3) Shakibi Nia, N.; Martucci, A.; Granozzi, G.; García, G.; Pastor, E.; Penner, S.; Bernardi, J.; Alonso-Vante, N.; Kunze-Liebhäuser, J. DEMS Studies of the Ethanol Electro-Oxidation on TiOC Supported Pt Catalysts–Support Effects for Higher CO2 Efficiency. *Electrochim. Acta* **2019**, *304*, 80–86. https://doi.org/10.1016/j.electacta.2019.02.089.

(4) Biesinger, M. C.; Lau, L. W. M.; Gerson, A. R.; Smart, R. S. C. Resolving Surface Chemical States in XPS Analysis of First Row Transition Metals, Oxides and Hydroxides: Sc, Ti, V, Cu and Zn. *Appl. Surf. Sci.* **2010**, *257* (3), 887–898. https://doi.org/10.1016/j.apsusc.2010.07.086.

(5) Frank, O.; Zukalova, M.; Laskova, B.; Kürti, J.; Koltai, J.; Kavan, L. Raman Spectra of Titanium Dioxide (Anatase, Rutile) with Identified Oxygen Isotopes (16, 17, 18). *Phys. Chem. Chem. Phys.* **2012**, *14* (42), 14567. https://doi.org/10.1039/c2cp42763j.

(6) Szabados, L.; Winkler, D.; Stock, D.; Alexander, T.; Lörting, T.; Kunze-Liebhäuser, J.; Portenkirchner, E. Sodium-Containing Surface Film Formation on Planar Metal–Oxide Electrodes with Potential Application for Sodium-Ion and Sodium–Oxygen Batteries. *Adv. Energy Sustain. Res.* **2022**, *3* (12), 2200104. https://doi.org/10.1002/aesr.202200104.
